# A Combined Infrared and Computational Study of Gas-Phase Mixed-Ligand Rhodium Complexes: Rh(CO)_*n*_(N_2_O)_*m*_^+^ (*n* = 1–5, *m* = 1–4)

**DOI:** 10.1021/acs.jpca.3c05078

**Published:** 2023-10-31

**Authors:** Gabriele Meizyte, Rachael H. Brown, Edward I. Brewer, Peter D. Watson, Stuart R. Mackenzie

**Affiliations:** Department of Chemistry, University of Oxford, Physical and Theoretical Chemistry Laboratory, South Parks Road, Oxford, United Kingdom, OX1 3QZ

## Abstract

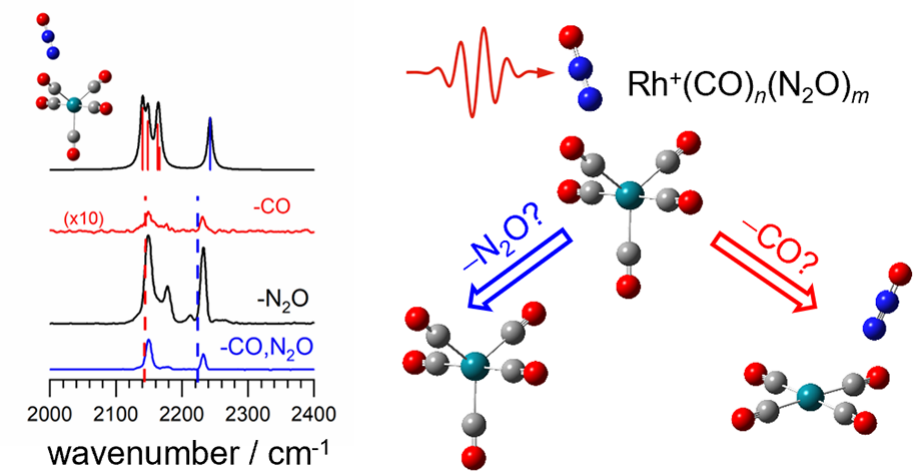

In this study, mixed carbonyl and nitrous oxide complexes with Rh^+^ were studied by mass-selective infrared photodissociation spectroscopy in a molecular beam. The infrared spectra, recorded in the region of the CO and N_2_O N=N stretches, were assigned and interpreted with the aid of simulated spectra of low-energy structural isomers. Clear evidence of an inner coordination shell of four ligands is observed. The observed vibrational structure can be understood on the basis of local mode vibrations in the two ligands. However, there is also evidence of multiple low-lying isomers and cooperative binding effects between the two ligands. In particular, σ donation from directly coordinated nitrous oxide ligands drives more classical carbonyl bonding than has been observed in pure carbonyl complexes. The observed fragmentation branching ratios following resonant infrared absorption are explained by simple statistical and energetic arguments, providing a contrast with those of equivalent Au^+^ complexes.

## I Introduction

The use of metal atoms and clusters in catalysis is commonplace. Such catalysts are often able to bind multiple chemical partners and foster the reactivity between them. In the case of heterogeneous catalysis, this idea is famously applied in the three-way catalytic converter, in which the reduction of nitrogen oxides (NO, N_2_O, etc.) occurs in parallel with the oxidation of CO.^1^ Highly dispersed rhodium plays a key role in this chemistry as the nitrogen oxide reduction catalyst,^2−4^ and this has prompted extensive work on NO_*x*_ binding to (and the reactivity with) gas-phase rhodium clusters.^5−8^

Even individual metal ions can support full catalytic cycles, as demonstrated by Kappes and Staley for Fe^+^ ions:
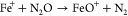

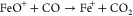
Several other single metal cations and small metal clusters have been shown to support similar catalytic cycles.^9^ However, similar experiments with Rh^+^ fail due to it reacting too slowly to complete the catalytic cycle at room temperature.^10,11^ The full cycle has been established on neutral Rh_*x*_ and Rh_*x*_O_*y*_ clusters, with the latter being more efficient.^12^ Charged rhodium clusters, Rh_*x*_^±^, exhibit size-dependent reactivity with both NO^5−7^ and N_2_O,^8^ and the infrared excitation of molecularly adsorbed Rh_*x*_N_2_O^+^ clusters induces a reaction and N_2_ loss.^13−16^ Rh_*x*_N_2_O^+^ clusters also support the full redox chemistry in reactions with CO.^17^

Infrared photodissociation (IR-PD) spectroscopy can provide very direct information about molecular binding, including molecular activation at metal centers. Thus, it represents a key technique for investigating the fundamental interactions underpinning catalytic reactions. IR-PD has been used previously to characterize the gas-phase ion−molecule complexes Rh^+^(CO)_*n*_ and Rh^+^(N_2_O)_*m*_ in detail.^18,19^ Here, we apply the same technique to explore the cooperative binding effects in mass-selected mixed-ligand complexes, Rh^+^(CO)_*n*_(N_2_O)_*m*_ (*n, m* = 1–4). These cooperative effects (i.e., the influence of one ligand binding on the binding of a second) can play important roles in catalysis, yet they remain far from understood.^20−22^

In addition to representing model systems for molecular activation, gas-phase mixed-ligand metal complexes provide a unique environment, wherein one can study the flow of vibrational energy across often complex potential energy landscapes. In a recent IR-PD study of Au(CO)_*n*_(N_2_O)_*m*_^+^ complexes, in addition to characterizing the structures, we reported intriguing non-ergodic fragmentation dynamics.^23^ A clear preference for CO binding directly to the Au^+^ ion was observed, with N_2_O being more weakly bound and often forced into a second solvation shell around a Au^+^(CO)_2_ or Au^+^(CO)_3_ core. CO and N_2_O were deliberately chosen as ligands because of their similar but distinct IR fundamental wavenumbers (C≡O stretches at 2143.2 cm^–1^ and N_2_O (N=N) stretches at 2223.5 cm^–1^).^24,25^ These provide spectrally addressable near-local modes into which known photon energies can be injected to study the fragmentation dynamics. As expected from the binding energy considerations, only N_2_O loss was observed for the smaller complexes. However, for all Au(CO)_*n*_(N_2_O)_*m*_^+^ (*n* ≥ 3) complexes, highly non-statistical fragmentation was observed, with the CO loss channel dominating the branching ratios despite CO being bound up to twice as strongly as N_2_O.^23^

Here, we present a combined infrared spectroscopic and computational study of gas-phase Rh(CO)_*n*_(N_2_O)_*m*_^+^ complexes, where we explore the simultaneous binding of CO and N_2_O on Rh^+^ and compare the fragmentation dynamics with those of Au(CO)_*n*_(N_2_O)_*m*_^+^.

CO binding to gas-phase Rh_*n*_^+/0/–^ clusters has been studied extensively by Fielicke and co-workers using free-electron laser infrared multiple-photon dissociation (IRMPD). In both Rh_*x*_CO^+/0/-^ (*x* = 3–15)^26,27^ and Rh_*x*_(CO)_*n*_^+/0^ (*x* = 1–6, *n* = the saturation limit),^28^ the CO binding motif was found to be strongly size- and charge-state-dependent.

Among the extensive IR-PD studies of metal ion−carbonyl complexes that have been completed, Duncan and co-workers reported “non-classical” binding in both Rh(CO)_*n*_^+^ and Au(CO)_*n*_^+^, characterized by a spectral blue-shift in the CO stretching frequency rather than the red-shift that is observed more commonly observed in binding to metal centers.^18,29^ Non-classical binding is typical of late transition metal−carbonyl complexes, in which filled *d* electron shells reduce both the efficiency of σ donation and π back-bonding.^30^ In Au(CO)_*n*_^+^ (*n* = 3–6), the carbonyl stretch is blue-shifted 50–100 cm^–1^ with respect to the free CO stretch.^18,29^ In Rh(CO)_*n*_^+^, the spectra are more complex, with both blue- and red-shifted bands being observed.^18,29^ Assignment is complicated by unpaired d electrons, which give rise to multiple low-lying spin states and unusual structural motifs.

Nitrous oxide binding to metal cations provides rich isomeric distributions, which arise from the possibility of spectrally distinguishable N and O binding motifs.^19,23,31,32^ Direct N binding to the metal ion occurs by σ donation, leading to a characteristic blue-shift in the N=N stretching mode of up to 200 cm^–1^; this clearly identifies N_2_O in the inner coordination shells. In addition to multiple isomers, IR-PD studies of Rh(N_2_O)_*m*_^+^ (*m* = 1–7) complexes have revealed the role of singlet and triplet electronic states, whose relative energies switch upon the binding of additional ligands.^19^ The spectra of Au(N_2_O)_*m*_^+^ complexes, by contrast, are comparatively simple, with single spectral bands present for N- and O-bound complexes, respectively.^31^ Excited Au^+^ electronic states may well be quenched by N_2_O, as suggested by Armentrout and co-workers.^33,34^

## II Experimental and Computational Methods

The experimental setup used here was the same as for the Au(CO)_*n*_(N_2_O)_*m*_^+^ study and has been described in detail elsewhere.^23,35,36^ Briefly, a rotating disc target of the Rh metal is ablated at 532 nm by the output of a pulsed Nd:YAG laser (10 Hz, 8 ns, ca. 2–10 mJ as necessary). Ablated atoms and ions cool and cluster by collision in a pulsed gas mixture of Ar seeded with N_2_O (5%) and CO (1% or 3%, depending on the species desired). Typical backing pressures were 4–6 bar. The gas mix, now entrained with a range of metal-containing species, expands into the vacuum, forming a molecular beam that is then skimmed. Charged complexes are mass-selected in a quadrupole mass filter/bender (QMF/B) assembly before their perpendicular extraction into a reflectron time-of-flight (ToF) mass spectrometer. The experiment runs at room temperature, but the internal energy distribution of the molecular beam has not been determined.

In order to record IR-PD spectra, alternate cluster beam pulses were subjected to infrared pulses from a tunable optical parametric oscillator/optical parametric amplifier (OPO/OPA) system (LaserVision) operating in the 2000–2400 cm^–1^ range. Following parent complex mass selection, spectra were recorded as a function of the wavenumber in the daughter fragment channels against a zero background. By measuring fragment yields as a function of the excitation wavenumber, we obtained infrared action spectra as well as fragmentation branching ratios for comparison with those simulated for calculated structures.

Energetically low-lying structures and any relevant transition states connecting them were calculated with the B3PW91^37,38^/def2TZVP^39,40^ functional/basis set combination with empirical dispersion GD3BJ^41^ using the Gaussian 16 software package.^42^ In addition to chemical intuition, novel potential structures were generated by a modified Kick algorithm.^43^ The results were tested by comparison with those obtained using the B3P86^37,44^/def2TZVP^39,40^ combination, which has been proven to be reliable in similar previous studies.^19,23,31^ To ease comparison with the experimental data, the computed harmonic vibrational frequencies were scaled by a factor of 0.965 and 0.933, which were determined by calculating the vibrational frequency of the free CO and N=N in the free N_2_O stretches, respectively.^24,25^ Computed infrared bands were convoluted with Lorentzian line widths with the full width at half-maximum (FWHM) = 8 cm^–1^. All energies reported here are given relative to the zero-point levels of the lowest-energy molecularly adsorbed structure. Only molecularly bound ligands were considered, as only these are expected to exhibit spectra in our experimental region. Dissociatively bound structures are calculated to lie substantially lower in energy but are kinetically inaccessible under our experimental conditions behind large activation barriers. Potential energy profiles were generated by identifying plausible transition states between minima and performing intrinsic reaction coordinate (IRC) calculations in order to verify the path.^45,46^

## III Results and Discussion

### A Time-of-Flight Mass Spectra

**Figure 1:**
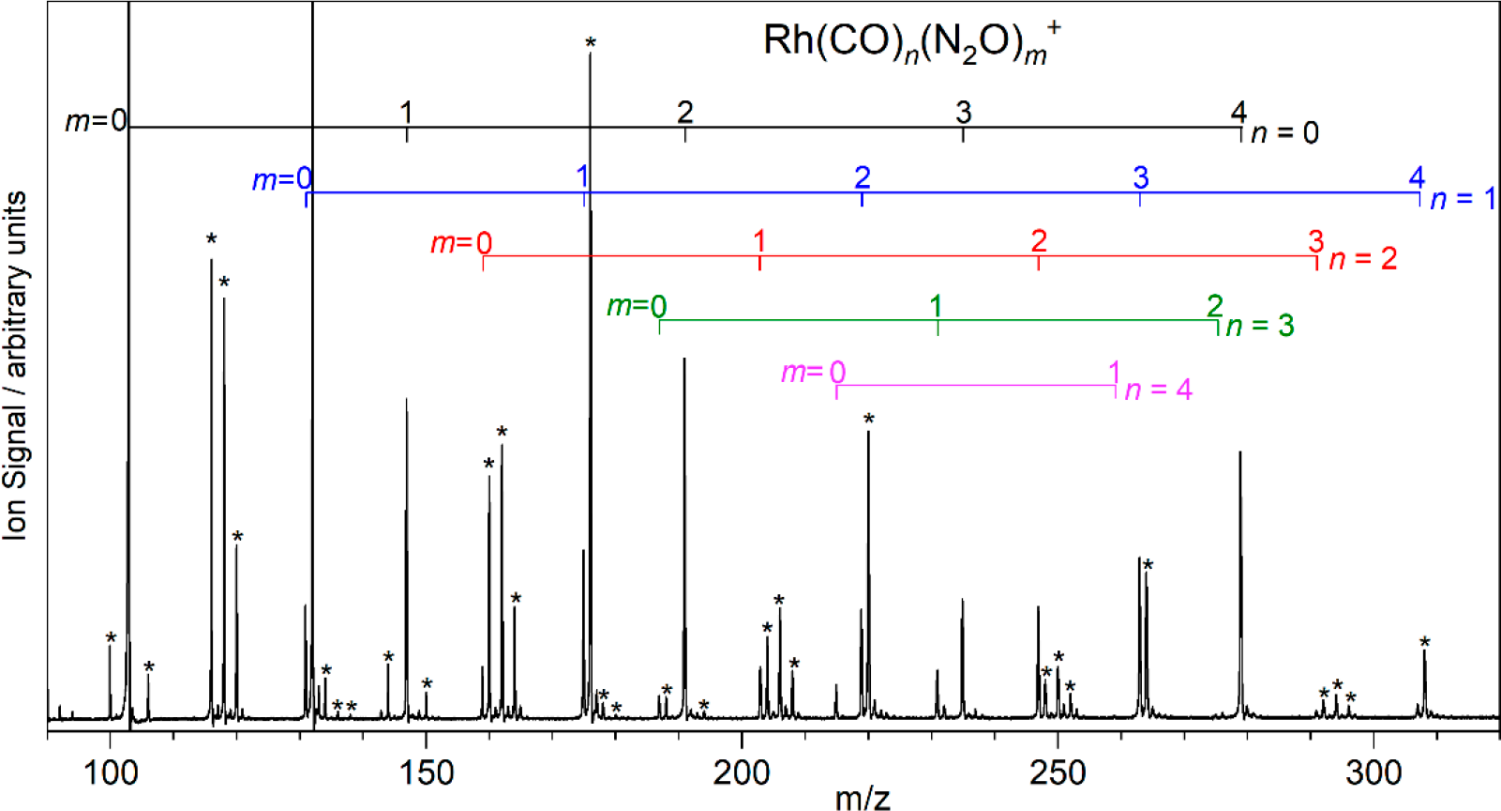
Time-of-flight mass spectrum of Rh(CO)_*n*_(N_2_O)_*m*_^+^ complexes produced by laser ablation of the Rh target in the presence of Ar seeded with 1% CO and 5% N_2_O. Assignments are grouped by the number of CO ligands: *n* = 0 (black), 1 (blue), 2 (red), 3 (green), and 4 (pink). Peaks with an asterisk have an even mass and do not contain Rh atoms.

Figure 1 shows a representative time-of-flight mass spectrum that illustrates the typical range of Rh(CO)_*n*_(N_2_O)_*m*_^+^ complexes that were generated. Signals are comparatively strong until a total (*n* + *m*) of four ligands are bound, after which the intensity drops significantly, which suggests that this represents a first complete coordination shell.

The mass spectrum is relatively clean with only minor reaction products, such as RhO_*y*_^+^. Other series of peaks (marked with asterisks) are observed, which are tentatively assigned to the molecular complexes [(CO)_*n*_(N_2_O)_*m*_]^+^, [NO(N_2_O)_*m*_]^+^, [O_2_(N_2_O)_*m*_]^+^, etc., on the basis of their even masses (i.e., they do not include Rh atoms). These are almost certainly produced when electrons liberated from the metal target ionize gas molecules, which then form nucleation sites for complexation.

### B Infrared Spectra of Rh(CO)_*n*_(N_2_O)_*m*_^+^ Ion–Molecule Complexes

**Figure 2:**
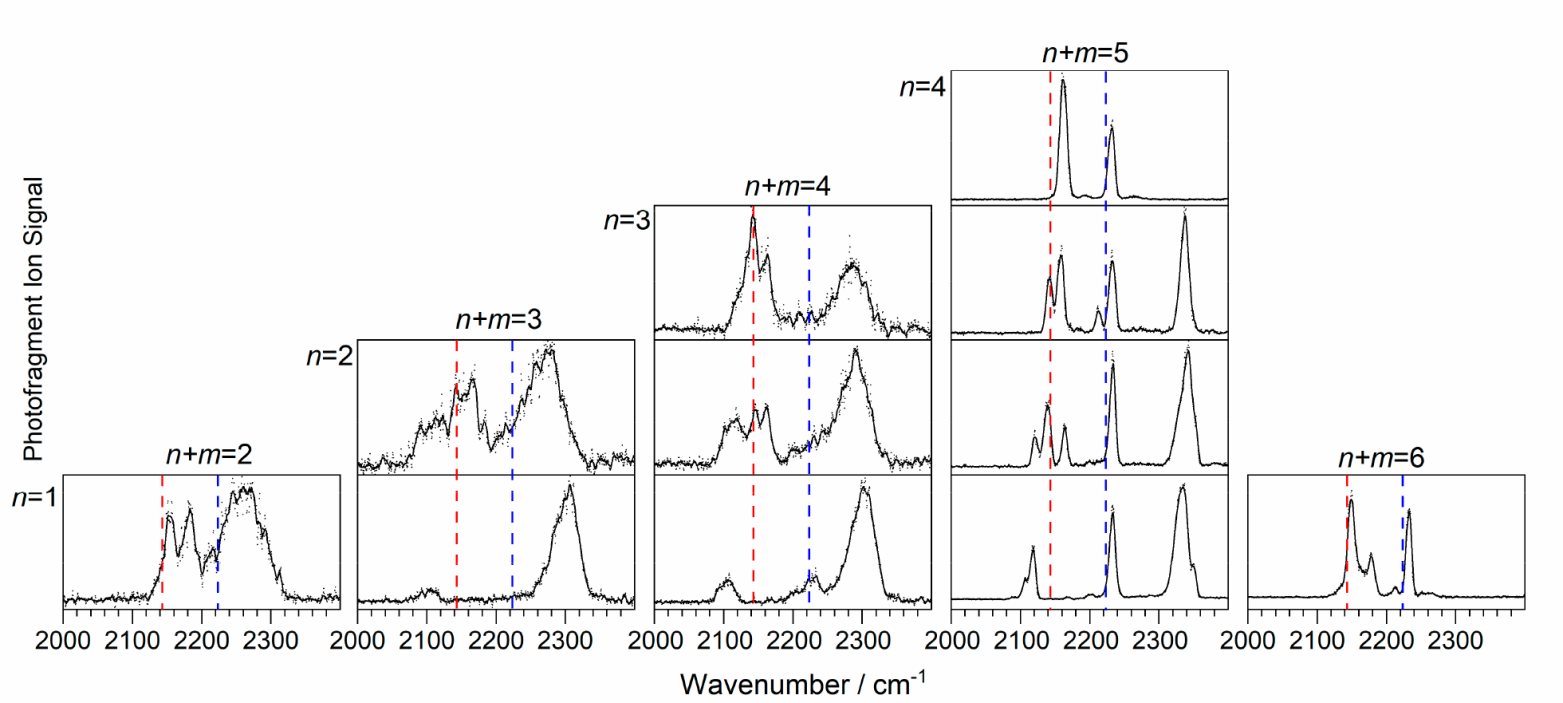
IR-PD spectra of Rh(CO)_*n*_(N_2_O)_*m*_^+^ (*n* = 1–5, *m* = 1–4) species recorded as the absolute enhancement signal in the respective N_2_O loss channels. Spectra are grouped in columns by the total number of ligands (*n* + *m*). The dashed red and blue lines denote the free CO (2143.2 cm^–1^) and N_2_O N=N (2223.5 cm^–1^) stretches, respectively.^24,25^

Figure 2 shows an overview of the IR-PD spectra of Rh(CO)_*n*_(N_2_O)_*m*_^+^ recorded in this study. To explore the trends, the spectra have been plotted with the total number of ligands (*n* + *m*) increasing from left to right and the number of CO ligands *n* increasing from bottom to top. Tabulated peak positions and comparisons with scaled calculated frequencies are provided in the Supporting Information (Table S2).

One immediate observation from Figure 2 is the marked change in the spectral line widths between smaller complexes (up to and including four ligands) and larger ones. The spectra of the smaller complexes are characterized by broad, partially resolved features that are typical of multiple-photon excitation and/or the presence of a distribution of structures (isomeric forms or electronic states). Multiple-photon dissociation is typical for higher binding energy complexes, and this would be consistent with an inner coordination shell of four ligands directly bound to the Rh^+^ ion. In principle, inert messenger (e.g., Ar) tagging could be used to generate better resolved spectra of the smaller complexes, thus yielding improved structural information. However, in our current experimental arrangement, the combination of Ar-tagging coupled with the low transmission of the quadrupole mass filter does not yield sufficient number densities for spectra with acceptable signal-to-noise ratios.

The spectra of the larger (*n* + *m* ≥ 5) complexes are considerably better resolved, having narrower bands that are consistent with single-photon absorption being sufficient for removing a weakly bound ligand from an outer coordination shell. This idea of an inner coordination shell of four is consistent with the cluster distribution observed in the time-of-flight mass spectra. The calculated binding energies for each of the complexes discussed are presented in Table S3 in the Supporting Information. These show the expected drop in binding energy when the second solvation shell opens. For *n* + *m* ≤ 4, the lowest binding energy (corresponding to N_2_O loss) is ca. 0.82 eV. Even accounting for the likely internal energy of a complex, this represents at least two (2260 cm^–1^ or 0.28 eV) IR photons in this spectral region. By contrast, an N_2_O ligand bound in the second solation shell (*n* + *m* ≥ 5) has a binding energy of 0.17 eV, making for facile loss at the one photon level.

The IR-PD spectra in Figure 2 were all recorded in the N_2_O loss channel, which is the dominant fragmentation channel for all of the complexes recorded here. Other loss channels (CO loss, [CO, N_2_O] loss, etc.) represent minor fragmentation pathways and are only observed for larger (*n* + *m* ≥ 5) complexes (see Sections C and D below as well as the Supporting Information). This marks a significant difference from our previous Au(CO)_*n*_(N_2_O)_*m*_^+^ study, in which CO loss dominated the fragmentation of many complexes. In the case of Rh^+^ complexes, CO is only ever lost from the outer coordination shell and is found preferentially bound directly to the metal ion center.

**Figure 3:**
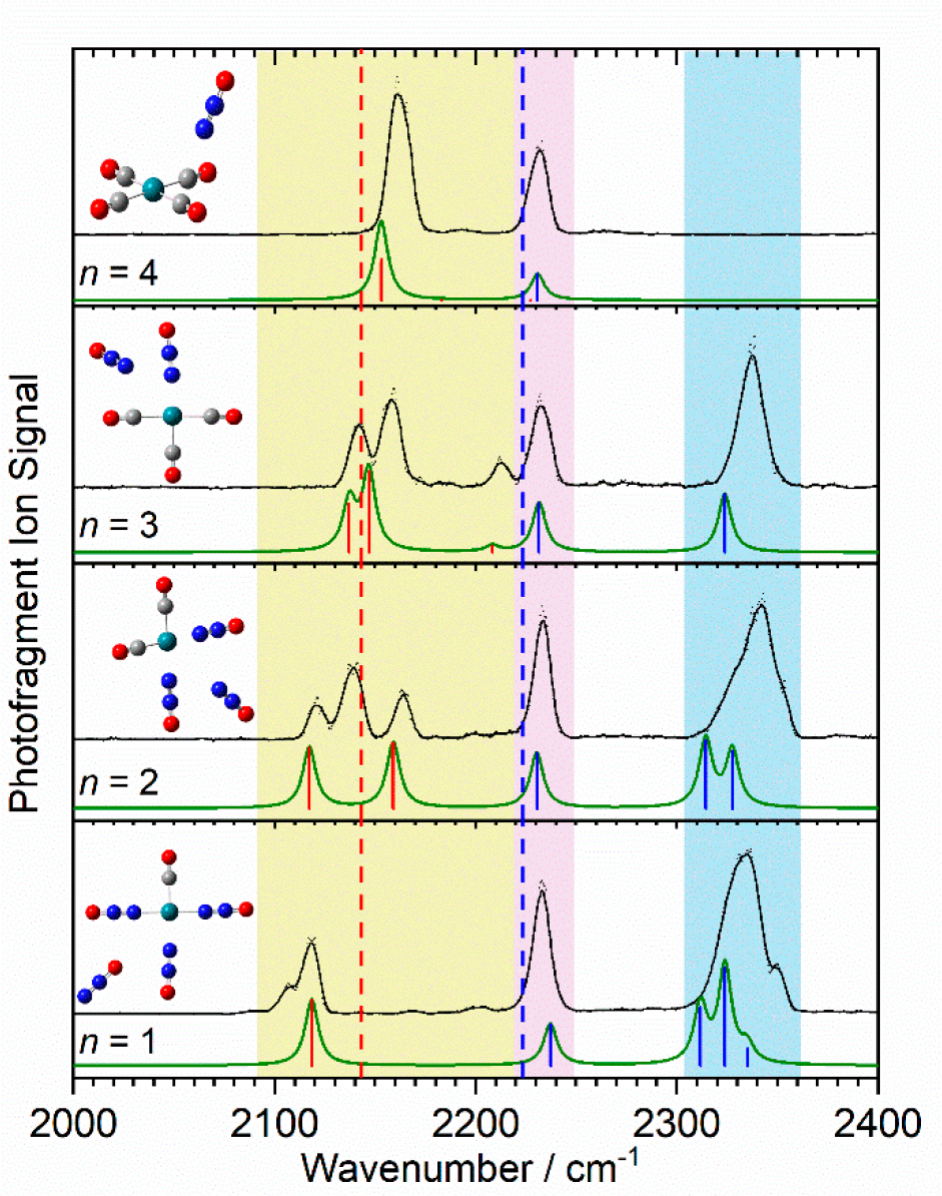
IR-PD spectra of the Rh(CO)_*n*_(N_2_O)_*m*_^+^ (*n* = 1–4, *n* + *m* = 5) complexes. In each case, the experimental spectrum is compared to the simulated spectrum based on the lowest-energy calculated structure that was obtained. The dashed red and blue lines denote the free CO (2143.2 cm^–1^) and N_2_O N=N (2223.5 cm^–1^) stretches, respectively.^24,25^ Colored bands represent common vibrations between species, as discussed in the text.

Given the cleaner nature of their spectra, the structural assignment of the larger Rh(CO)_*n*_(N_2_O)_*m*_^+^ complexes is more straightforward, as shown in Figure 3 for the complexes with a total of five ligands. Vibrational bands are observed in three spectrally characteristic regions. In the region of 2090–2220 cm^–1^ (yellow), the bands are confidently assigned to CO stretches. In the case of the Rh(CO)_*n*_(N_2_O)_*m*_^+^ (*n* = 1 and 2) complexes, weakly red-shifted bands are observed. This classical carbonyl binding is a cooperative effect that is consistent with the electron density donated by the σ binding of the N_2_O ligands stabilizing the positive charge on Rh^+^, thus promoting π* back-bonding.^18^ These bands shift further to the blue as the CO:N_2_O fraction increases (bottom to top in Figure 3) as this effect reduces. In the Rh(CO)_2_(N_2_O)_3_^+^ and Rh(CO)_3_(N_2_O)_2_^+^ spectra, more CO bands are observed as a result of in- and out-of-phase combinations of CO stretches. In Rh(CO)_4_(N_2_O)^+^, the N_2_O ligand occupies the second coordination shell and essentially acts as a messenger species. The single intense feature at 2160 cm^–1^ (pairwise out-of-phase CO stretches) is identical to that of the non-classical square planar Rh(CO)_4_^+^ core reported by Brathwaite and co-workers.^18^

The remaining bands present in Figure 3 (the pink and blue regions) represent characteristic N=N stretching modes. The pink band around 2235 cm^–1^ (i.e., close to the free N_2_O band) originates from weakly perturbed N_2_O molecule(s) bound in a secondary solvation shell. The 2320–2350 cm^–1^ band (the blue region) is assigned to N-bound N_2_O ligands that are bound directly to the Rh^+^ ion. σ donation from the HOMO-1 orbital leads to the observed significant (ca. 120 cm^–1^) blue-shift in the N=N stretch. This feature is absent in the spectrum of the Rh(CO)_4_(N_2_O)^+^ complex, as the lone N_2_O ligand occupies the outer shell and the same σ donation is not possible. The spectra in this region are simpler than those observed previously for the bare Rh(N_2_O)_*m*_^+^ complexes, whose assignment invoked the presence of multiple electronic states.^31^ Equally, there is no evidence of O-bound N_2_O in any of our spectra. These would be spectrally indistinguishable in the red region, but direct O binding to the Rh^+^ leads to much higher energy isomeric structures.

**Figure 4:**
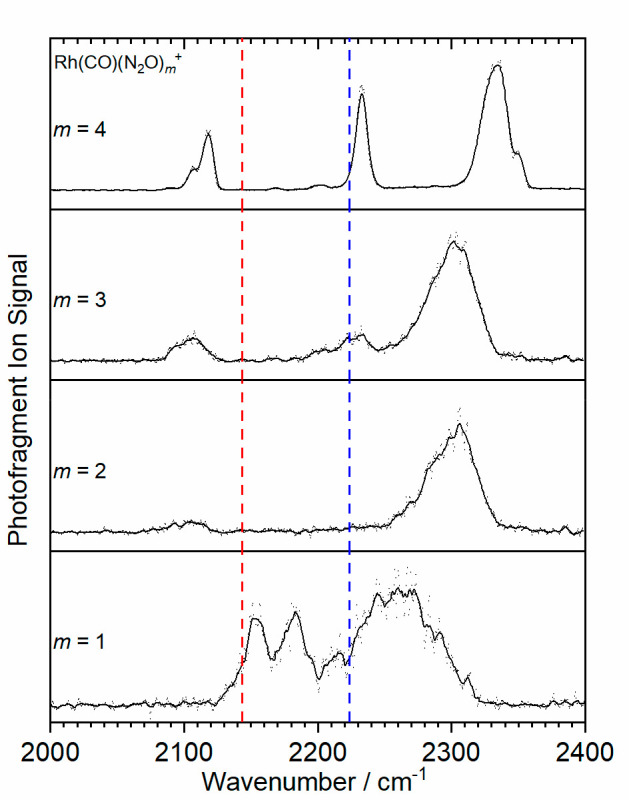
IR-PD spectra of the Rh(CO)(N_2_O)_*m*_^+^ (*m* = 1–4) complexes recorded in the N_2_O loss channel. The dashed red and blue lines denote the free CO (2143.2 cm^–1^) and N_2_O N=N stretches (2223.5 cm^–1^), respectively.^24,25^

Figure 4 shows a different trend in the infrared spectra, namely that of an increasing number of N_2_O ligands to a Rh(CO)^+^ core. These Rh(CO)(N_2_O)^+^ (*m* = 1–4) spectra exhibit many of the same features as in Figure 3 with the carbonyl and nitrous oxide bands distinguishable. In the Rh(CO)(N_2_O)^+^ complex, two CO stretch bands are partially resolved at 2153 and 2181 cm^–1^, suggesting the presence of two low-lying structural isomers that both exhibit poor π back-bonding with limited charge donation from the single N_2_O.

As the number of nitrous oxide ligands increases, two notable changes are observed. First, increased σ donation results in the emergence of classical carbonyl binding signified by the red-shift from the free CO band. Simultaneously, the N_2_O band first blue-shifts and then splits into the now familiar two bands corresponding to first (2330–50 cm^–1^) and second (2233 cm^–1^) coordination shell binding, with all bands becoming markedly narrower once the second shell opens (*n* + *m* = 5). Similar trends were observed by Cunningham et al. in their Ar-tagged IR-PD spectra of Rh(N_2_O)_*m*_^+^, which had spectral features in the 2240–2320 cm^–1^ region assigned to the presence of triplet states.^19^ They calculated that triplet states form the ground states for the *m* < 3 complexes, but the singlet state lies lower in energy for larger complexes. Perhaps unsurprisingly, where mixed ligands are found in the same coordination shell, there is evidence (in terms of partially resolved vibrational bands) of multiple isomeric structures being present.

In order to better understand the trends observed in the experimental spectra and to confirm the assignments given, we compared the experimental spectra with those simulated for low-energy structures calculated using density functional theory.

### C Comparison of Experimental and Simulated Spectra

**Figure 5:**
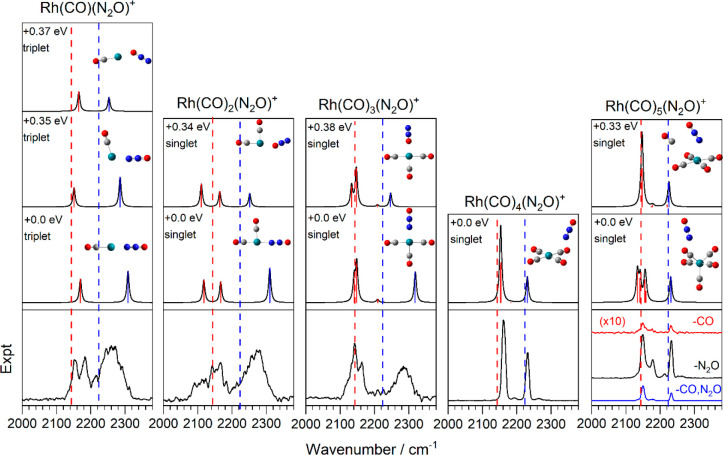
Comparison of the experimental and simulated spectra of the Rh(CO)_*n*_(N_2_O)^+^ (*n* = 1–5) complexes. Spectra are presented as enhancements in the N_2_O loss channel unless otherwise indicated. The band positions in red are CO modes, while N_2_O vibrations are in blue. The dashed red and blue lines denote the experimental values of free CO (2143.2 cm^–1^) and N_2_O N=N (2223.5 cm^–1^) stretches, respectively.^24,25^

Figure 5 shows a comparison of the experimental action spectra of Rh(CO)_1–5_(N_2_O)^+^ complexes to those predicted for the energetically lowest-lying calculated structures. Similar comparisons of the experimental and calculated spectra for Rh(CO)(N_2_O)_1–4_^+^ and Rh(CO)_2,3_(N_2_O)_2,3_^+^ are provided in Figures S1 and S2 of the Supporting Information. With the exception of the Rh(CO)(N_2_O)^+^ complex, all of the low-energy states were calculated to be singlet states. The CO molecule binds exclusively via the C atom, and all ground states show N binding of the nitrous oxide. O-bound isomers typically form excited structural forms that are approximately 0.35 eV higher in energy, which is consistent with the work of Cunningham et al.^19^

There is evidence of the presence of excited isomeric forms in both the broad line width and the partially resolved structure in the spectra of the Rh(CO)_1–3_(N_2_O)^+^ complexes. In the high collision environment of the cluster source, it is possible to trap the excited structural forms of small complexes behind kinetic barriers. For example, the barrier of internal N_2_O rotation in Rh(CO)(N_2_O)^+^ is calculated to be 0.82 eV. Thus, although the thermodynamic ground state contains N-bound N_2_O, O-bound structures, once formed, cannot access the global minimum. Each structural motif gives rise to a different vibrational spectrum, with O-bound N_2_O vibrations found closer to the free N_2_O stretch and presenting negligible perturbation to the N=N stretch. By contrast, N_2_O stretches in the N-bound isomers are strongly blue-shifted from the free N_2_O value due to the donation from the 7σ orbital.

There is clear evidence for both classical and non-classical carbonyl binding in the smaller complexes, and indeed, the spectra are very similar in the CO stretch region to those reported by Brathwaite et al. for pure Rh(CO)_*n*_^+^ complexes.^18^ Likewise, the square planar structure of the Rh(CO)_4_^+^ core is reproduced here, as is the square-based pyramidal structure of the Rh(CO)_5_^+^ core with its unique axial ligand.^18^ The nitrous oxide in our case essentially plays the role of the messenger.

The Rh(CO)_5_(N_2_O)^+^ spectrum exhibits fragmentation into the −N_2_O, −CO and −(CO, N_2_O) channels, with a branching ratio of approximately 0.8:0.18:0.02. The predominance of N_2_O loss is reflected in its (weak) binding in the outer coordination shell. CO loss may arise because the axial CO is more weakly bound than the others,^18^ or it may signify the presence of the excited isomeric form, in which CO binds in the outer shell. Typically, d^8^ metals with 16 valence electrons prefer square planar geometries, as observed for Rh(CO)_4_^+^ and Rh(N_2_O)_4_^+^,^18,19^ but then break planarity at 18 valence electrons,^47^ as is the case here.

### D Comparison between Rh(CO)_*n*_(N_2_O)*_m_*^+^ and Au(CO)*_n_*(N_2_O)*_m_*^+^ Complexes

One of the principal reasons for studying the spectra and fragmentation dynamics of Rh(CO)_*n*_(N_2_O)_*m*_^+^ was to compare the results with our previous results on the equivalent Au(CO)_*n*_(N_2_O)_*m*_^+^ complexes. It is clear that replacing Au^+^ with Rh^+^ leads to substantially different complex structures and fragmentation dynamics.^23^

Ground-state Rh^+^ has a d^8^ electronic configuration, giving rise to low-lying triplet excited states (Figures 5, S1, and S2 and the Supporting Information). By contrast d^10^ Au^+^ has only low-lying singlet states, with triplet states typically 3 eV higher in energy.^23^ Both Au^+^ and Rh^+^ complexes exhibit non-classical carbonyl binding, and the CO blue-shifts observed in the rhodium complexes are much smaller than those observed in the gold complexes.

There are also key differences in the geometrical structures of the Au^+^ and Rh^+^ complexes. Famously, linear Au(L)_2_^+^ (L = ligand) complexes are stabilized by the Orgel effect,^30^ in which the effective hybridization of the full 6d^10^ shell with the empty 5s shell leads to strong polarization. Such effects are absent in Rh^+^, which instead exhibits a clear coordination shell of four ligands. The result is that N_2_O remains tightly bound directly to Rh^+^ as long as there is a vacancy in the inner coordination shell (i.e., *n* < 4). In contrast, in the case of Au^+^, even two CO ligands are sufficient to displace N_2_O into a secondary coordination shell with profound consequences for the binding energies. In the smallest complexes, the binding of both ligands to Au^+^ is stronger than that to Rh^+^: the calculated CO and N_2_O binding energies in Au(CO)(N_2_O)^+^ are 2.56 and 1.67 eV, respectively, while those in Rh(CO)(N_2_O)^+^ are 1.94 and 1.25 eV, respectively. These greatly exceed the IR photon energy, and thus, their action spectra are not observed. However, a single additional CO (or N_2_O) ligand to Au(CO)(N_2_O)^+^ is enough to reduce the N_2_O binding energy to 0.14 eV (0.12 eV), leading to efficient loss upon infrared absorption and sharp spectral features. In contrast, the N_2_O binding energy in Rh(CO)(N_2_O)_2_^+^ is >0.75 eV (>1.25 eV in Rh(CO)_2_(N_2_O)^+^) and only falls below 0.75 eV once a second coordination shell opens with a fifth ligand.

**Figure 6:**
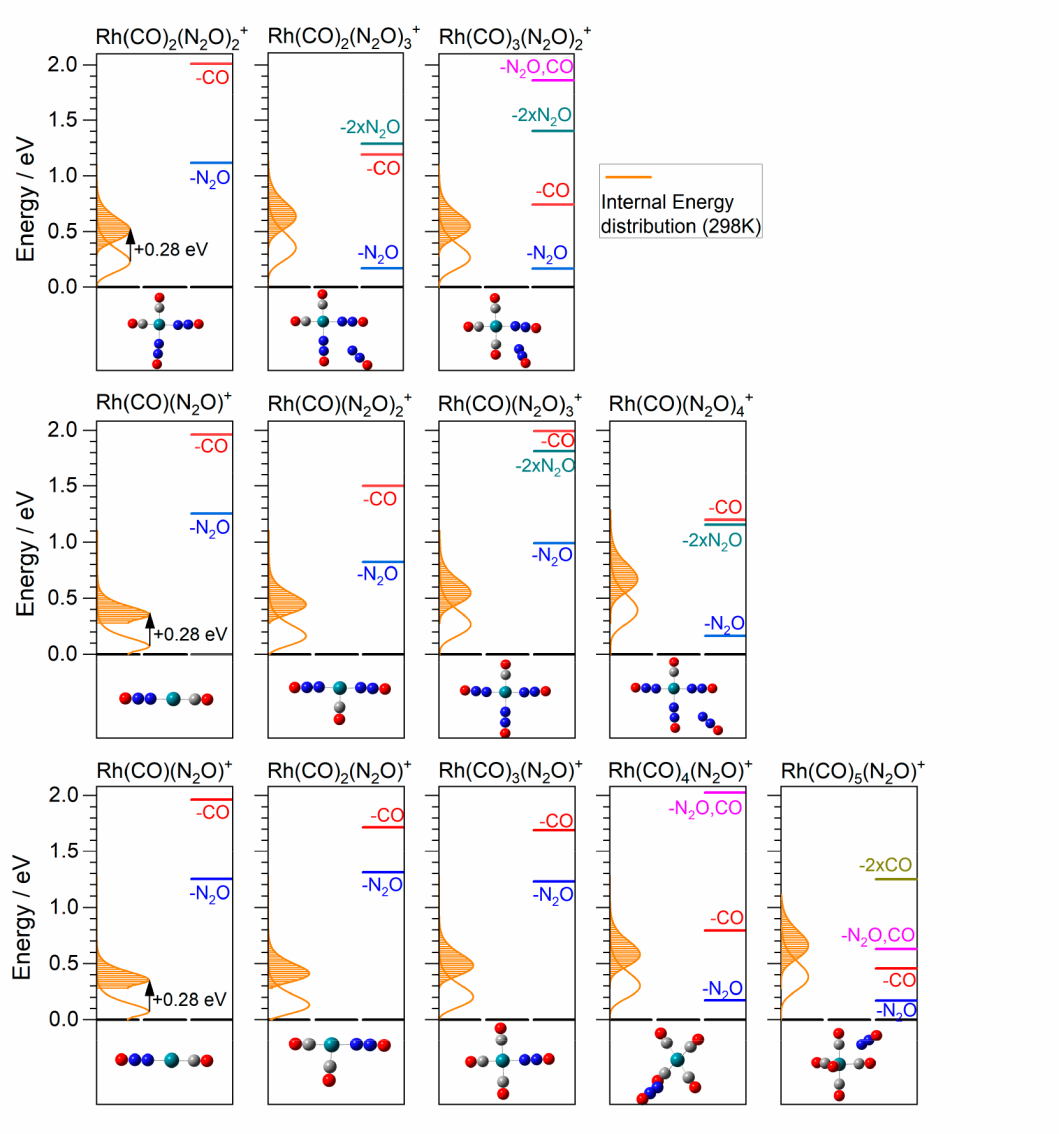
Calculated internal energy distributions for the putative ground-state structures of various Rh(CO)_*n*_(N_2_O)_*m*_^+^ complexes at 298 K. The bottom, middle, and top rows show Rh(CO)_*n*_(N_2_O)^+^, Rh(CO)(N_2_O)_*m*_^+^, and assorted *n, m* = 2, 3 complexes, respectively. Shaded regions indicate the energies accessible following single-photon absorption at 2260 cm^–1^ (0.28 eV). Calculated fragmentation thresholds (the ligand binding energies) are also indicated.

It is helpful to estimate the calculated internal energy distributions and compare them with the calculated ligand binding energies. We adopt a similar approach as in ref (23) for Au(CO)_*n*_(N_2_O)_*m*_^+^ and determine the mean internal energy of the Rh^+^ complexes with respect to their zero-point energy:
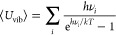
where the sum is over all vibrational modes and the other symbols have their usual meaning. The corresponding vibrational energy distribution as a function of energy *E* and temperature *T* takes the form
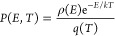
where *q*(*T*) is the total molecular partition function and ρ(*E*) is the vibrational density of states calculated using the Beyer–Swinehart algorithm^48^ in the MultiWell software.^49^ Our cluster beam is very unlikely to be thermalized. The actual internal energy distribution results from a complex mix of, among other factors, size- and state-dependent collisional energy transfer, exothermic bond formation, and radiative decay. For indicative purposes only, we assume a vibrational temperature of 298 K (i.e., negligible cooling in the molecular beam). Figure 6 shows the calculated internal energy distributions for a range of Rh(CO)_*n*_(N_2_O)_*m*_^+^ complexes together with relevant dissociation thresholds (for N_2_O or CO loss). Additional internal energy plots that assume cooling to 200 K are provided in the Supporting Information (Figure S3).

It is clear from Figure 6 why only the larger complexes exhibit clean, well-resolved IR-PD spectra. All of the small (≤4 ligand) complexes require multiple-photon absorption to reach any fragmentation threshold, the lowest of which lies at ca. 0.80 eV. Only when the total number of ligands exceeds four do any fragmentation thresholds fall within reach of single-photon absorption. In the case of five ligands, only the N_2_O loss thresholds can be accessed by single-photon absorption; the nitrous oxide ligand, which is forced to occupy the outer solvation shell, essentially acts like a messenger species. The three fragmentation channels observed in Figure 5 for Rh(CO)_5_(N_2_O)^+^ are all accessible at the single photon level. In short, both the qualitative understanding of the spectra and the resulting fragmentation dynamics can be explained in terms of the dissociation thresholds relative to the calculated internal energy distributions. There is no evidence of anything other than simple statistical fragmentation with the weakest intermolecular bonds breaking.

The simple, predictable fragmentation dynamics of Rh(CO)_*n*_(N_2_O)_*m*_^+^ complexes, which are dominated by the loss of the most weakly bound ligand (N_2_O), contrasts markedly with the unusual non-ergodic nature of the dynamics reported previously in the case of Au(CO)_*n*_(N_2_O)_*m*_^+^. In that case, CO loss dominated the branching ratios for all complexes with *n* > 2, even though CO was bound up to twice as strongly compared to N_2_O.^23^ It seems increasingly likely that the differences in the behavior of the two complexes are due to a combination of vibrational coupling and hindered intramolecular vibrational energy redistribution (IVR), as invoked recently in the infrared dissociation spectra of the bispyridinium triflate ion pair.^50^ In the Au(CO)_*n*_(N_2_O)_*m*_^+^ (*n* ≥ 3) complexes, we suspect that the vibrational coupling between the N_2_O stretch, Au···N_2_O stretch, and Au(CO)_3_ wagging modes allows for efficient IVR into the pseudoplanar Au(CO)_3_ core, where energy becomes trapped. The in-plane CO stretch combination, in contrast, couples poorly to the perpendicular Au^+^···N_2_O mode, thus locking vibrational energy within the core and leading to CO fragmentation. Although this explanation can account qualitatively for the observations, a full quantum dynamics study is required to better understand the branching ratios.

In neither the Rh(CO)_*n*_(N_2_O)_*m*_^+^ nor Au(CO)_*n*_(N_2_O)_*m*_^+^ mixed-ligand systems do we observe any evidence for infrared-driven intracluster reactivity, as has been previously seen on metal clusters.^13,15−17,51−53^ When spectra are recorded in multiple dissociation channels, the spectra are similar in each fragmentation channel. With the possible exception of the combined −(CO, N_2_O) loss channel in some Au(CO)_*n*_(N_2_O)_*m*_^+^ complexes,^23^ there is little evidence of mode-selective fragmentation (e.g., enhanced CO loss following the pumping of a CO vibrational mode), suggesting there is efficient energy redistribution around the complex prior to dissociation.

## IV Summary and Conclusions

In summary, infrared action spectra of the Rh(CO)_*n*_(N_2_O)_*m*_^+^ (*n* = 1–5, *m* = 1–4) complexes have been recorded in the region of the C≡O and N=N N_2_O fundamental bands and have been assigned by comparison with simulated spectra of calculated low-energy structures. Clear evidence of a coordination shell closing at four ligands was observed. The spectra of the complexes smaller than this are characterized by broad spectral line widths and partially resolved vibrational structures that are typical of multiple-photon dissociation and the presence of different structural isomers, especially N- and O-bound N_2_O. These results were supported by calculations of the internal energy distributions and fragmentation thresholds.

The spectra of the larger complexes were typically well-resolved and interpreted in terms of non-classical CO binding, σ donation from N_2_O ligands, and cooperative binding effects between the two ligands. These were well-reproduced by the simulated spectra and understood based on the spectra of previous studies of isolated Rh(CO)_*n*_^+^ and Rh(N_2_O)_*m*_^+^ complexes.^18,19^

The fragmentation dynamics of Rh(CO)_*n*_(N_2_O)_*m*_^+^ implied by mass-resolved infrared photofragmentation branching ratios appear statistical and are dominated by the loss of the most weakly bound ligand, N_2_O. This contrasts with the fragmentation of some Au(CO)_*n*_(N_2_O)_*m*_^+^ complexes reported previously, which exhibited markedly non-ergodic fragmentation with unexpectedly efficient CO loss.

## Supporting Information Available

Computational details and geometry files of the identified low-energy structures, tabulated experimental and calculated line positions, comparisons of the experimental and simulated spectra, and the calculated fragmentation thresholds and internal energy distributions (PDF)

## References

[ref1] Kašpar J.; Fornasiero P.; Hickey N. Automotive catalytic converters: current status and some perspectives. Catal. Today 2003, 77 (4), 419–449. 10.1016/S0920-5861(02)00384-X.

[ref2] MCCABE R Steady-state kinetics of the CO-N_2_O reaction over an alumina-supported rhodium catalyst. J. Catal. 1990, 121 (2), 422–431. 10.1016/0021-9517(90)90250-N.

[ref3] Su Q.; Li Y.; Wang S.; Gao C. Detailed Mechanism for Reduction of N_2_O over Rhodium by CO in Automotive Exhaust. Top. Catal. 2013, 56 (1), 345–351. 10.1007/s11244-013-9978-4.

[ref4] Dann T. W.; Schulz K. H.; Mann M.; Collings M. Supported rhodium catalysts for nitrous oxide decomposition in the presence of NO, CO_2_, SO_2_ and CO. Appl. Catal., B 1995, 6 (1), 1–10. 10.1016/0926-3373(95)00006-2.

[ref5] Anderson M. L.; Ford M. S.; Derrick P. J.; Drewello T.; Woodruff D. P.; Mackenzie S. R. Nitric oxide decomposition on small rhodium clusters, Rh_*n*_^±^. J. Phys. Chem. A 2006, 110 (38), 10992–11000. 10.1021/jp062178z. 16986831

[ref6] Bakker J. M.; Mafuné F. Zooming in on the initial steps of catalytic NO reduction using metal clusters. Phys. Chem. Chem. Phys. 2022, 24 (13), 7595–7610. 10.1039/D1CP05760J. 35297928 PMC8966623

[ref7] Ford M. S.; Anderson M. L.; Barrow M. P.; Woodruff D. P.; Drewello T.; Derrick P. J.; Mackenzie S. R. Reactions of nitric oxide on Rh_6_^+^ clusters: abundant chemistry and evidence of structural isomers. Phys. Chem. Chem. Phys. 2005, 7 (5), 975–980. 10.1039/b415414b. 19791388

[ref8] Harding D.; Ford M. S.; Walsh T. R.; Mackenzie S. R. Dramatic size effects and evidence of structural isomers in the reactions of rhodium clusters, Rh_n_^+/^-, with nitrous oxide. Phys. Chem. Chem. Phys. 2007, 9 (17), 2130–2136. 10.1039/B618299B. 17464394

[ref9] Kappes M. M.; Staley R. H. Gas-phase oxidation catalysis by transition-metal cations. J. Am. Chem. Soc. 1981, 103 (5), 1286–1287. 10.1021/ja00395a080.

[ref10] Blagojevic V.; Orlova G.; Bohme D. K. O-atom transport catalysis by atomic cations in the gas phase: reduction of N_2_O by CO. J. Am. Chem. Soc. 2005, 127 (10), 3545–3555. 10.1021/ja044950m. 15755176

[ref11] Rondinelli F.; Russo N.; Toscano M. On the Pt^+^ and Rh^+^ Catalytic Activity in the Nitrous Oxide Reduction by Carbon Monoxide. J. Chem. Theory Comput. 2008, 4 (11), 1886–1890. 10.1021/ct800199b. 26620332

[ref12] Yamada A.; Miyajima K.; Mafune F. Catalytic reactions on neutral Rh oxide clusters more efficient than on neutral Rh clusters. Phys. Chem. Chem. Phys. 2012, 14 (12), 4188–4195. 10.1039/c2cp24036j. 22354062

[ref13] Hamilton S. M.; Hopkins S.; Harding D. J.; Walsh T. R.; Gruene P.; Haertelt M.; Fielicke A.; Meijer G.; Mackenzie S. R. Infrared Induced Reactivity on the Surface of Isolated Size-Selected Clusters: Dissociation of N2O on Rhodium Clusters. J. Am. Chem. Soc. 2010, 132, 1448–1449. 10.1021/ja907496c. 20078040

[ref14] Hermes A. C.; Hamilton S. M.; Hopkins W. S.; Harding D. J.; Kerpal C.; Meijer G.; Fielicke A.; Mackenzie S. R. Effects of Coadsorbed Oxygen on the Infrared Driven Decomposition of N_2_O on Isolated Rh_5_^+^ Clusters. J. Phys. Chem. Lett. 2011, 2 (24), 3053–3057. 10.1021/jz2012963.

[ref15] Parry I. S.; Kartouzian A.; Hamilton S. M.; Balaj O. P.; Beyer M. K.; Mackenzie S. R. Chemical Reactivity on Gas-Phase Metal Clusters Driven by Blackbody Infrared Radiation. Angew. Chem., Int. Ed. 2015, 54 (4), 1357–1360. 10.1002/anie.201409483. 25475369

[ref16] Hamilton S. M.; Hopkins W. S.; Harding D. J.; Walsh T. R.; Haertelt M.; Kerpal C.; Gruene P.; Meijer G.; Fielicke A.; Mackenzie S. R. Infrared-induced reactivity of N_2_O on small gas-phase rhodium clusters. J. Phys. Chem. A 2011, 115 (12), 2489–2497. 10.1021/jp201171p. 21391545

[ref17] Parry I. S.; Kartouzian A.; Hamilton S. M.; Balaj O. P.; Beyer M. K.; Mackenzie S. R. Collisional Activation of N_2_O Decomposition and CO Oxidation Reactions on Isolated Rhodium Clusters. J. Phys. Chem. A 2013, 117 (36), 8855–8863. 10.1021/jp405267p. 23941584

[ref18] Brathwaite A. D.; Abbott-Lyon H. L.; Duncan M. A. Distinctive Coordination of CO vs N_2_ to Rhodium Cations: An Infrared and Computational Study. J. Phys. Chem. A 2016, 120 (39), 7659–7670. 10.1021/acs.jpca.6b07749. 27627059

[ref19] Cunningham E. M.; Gentleman A. S.; Beardsmore P. W.; Mackenzie S. R. Structural isomers and low-lying electronic states of gas-phase M^+^(N_2_O)_*n*_ (M = Co, Rh, Ir) ion–molecule complexes. Phys. Chem. Chem. Phys. 2019, 21 (26), 13959–13967. 10.1039/C8CP05995K. 30417903

[ref20] Lee J. K.; Kung M. C.; Kung H. H. Cooperative Catalysis: A New Development in Heterogeneous Catalysis. Top. Catal. 2008, 49 (3–4), 136–144. 10.1007/s11244-008-9087-y.

[ref21] van der Vlugt J. I. Cooperative Catalysis with First-Row Late Transition Metals. Eur. J. Inorg. Chem. 2012, 2012 (3), 363–375. 10.1002/ejic.201100752.

[ref22] Wodrich M. D.; Hu X. Natural inspirations for metal-ligand cooperative catalysis. Nature Reviews Chemistry 2017, 2, 0099 10.1038/s41570-017-0099.

[ref23] Green A. E.; Brown R. H.; Meizyte G.; Mackenzie S. R. Spectroscopy and Infrared Photofragmentation Dynamics of Mixed Ligand Ion–Molecule Complexes: Au(CO)_x_(N2O)_y_^+^. J. Phys. Chem. A 2021, 125 (33), 7266–7277. 10.1021/acs.jpca.1c05800. 34433267

[ref24] Herzberg G. In Molecular Spectra and Molecular Structure: Spectra of Diatomic Molecules; R.E. Krieger Pub. Co., 1989; Vol 1.

[ref25] Herzberg G. In Molecular Spectra and Molecular Structure: Infrared and Raman Spectra of Polyatomic Molecules; R.E. Krieger Pub. Co., Malabar, Florida, U.S., 1991; Vol 2.

[ref26] Fielicke A.; von Helden G.; Meijer G.; Simard B.; Denommee S.; Rayner D. M. Vibrational spectroscopy of CO in gas-phase rhodium cluster-CO complexes. J. Am. Chem. Soc. 2003, 125 (37), 11184–11185. 10.1021/ja036897s. 16220925

[ref27] Fielicke A.; von Helden G.; Meijer G.; Pedersen D. B.; Simard B.; Rayner D. M. Size and charge effects on the binding of CO to small isolated rhodium clusters. J. Phys. Chem. B 2004, 108 (38), 14591–14598. 10.1021/jp049214j.

[ref28] Swart I.; de Groot F. M. F.; Weckhuysen B. M.; Rayner D. M.; Meijer G.; Fielicke A. The effect of charge on CO binding in rhodium carbonyls: From bridging to terminal CO. J. Am. Chem. Soc. 2008, 130 (7), 2126 10.1021/ja0772795. 18225899

[ref29] Velasquez J.; Njegic B.; Gordon M. S.; Duncan M. A. IR photodissociation spectroscopy and theory of Au^+^(CO)_n_ complexes: nonclassical carbonyls in the gas phase. J. Phys. Chem. A 2008, 112 (9), 1907–1913. 10.1021/jp711099u. 18266347

[ref30] Orgel L. E. 843. Stereochemistry of metals of the B sub-groups. Part I. Ions with filled d-electron shells. J. Chem. Soc. 1958, 4186–4190. 10.1039/jr9580004186.

[ref31] Cunningham E. M.; Gentleman A. S.; Beardsmore P. W.; Iskra A.; Mackenzie S. R. Infrared Signature of Structural Isomers of Gas-Phase M^+^(N_2_O)_n_ (M = Cu, Ag, Au) Ion–Molecule Complexes. J. Phys. Chem. A 2017, 121 (40), 7565–7571. 10.1021/acs.jpca.7b07628. 28925700

[ref32] Cunningham E. M.; Gentleman A. S.; Beardsmore P. W.; Mackenzie S. R. Infrared spectroscopy of closed s-shell gas-phase M^+^(N_2_O)_*n*_ (M = Li, Al) ion–molecule complexes*. Mol. Phys. 2019, 117 (21), 2990–3000. 10.1080/00268976.2019.1595202.

[ref33] Li F.-X.; Armentrout P. B. Activation of methane by gold cations: Guided ion beam and theoretical studies. J. Chem. Phys. 2006, 125 (13), 133114 10.1063/1.2220038. 17029440

[ref34] Li F.-X.; Gorham K.; Armentrout P. B. Oxidation of Atomic Gold Ions: Thermochemistry for the Activation of O_2_ and N_2_O by Au^+^ (^1^S_0_ and ^3^D). J. Phys. Chem. A 2010, 114 (42), 11043–11052. 10.1021/jp100566t. 20307075

[ref35] Brewer E. I.; Green A. E.; Gentleman A. S.; Beardsmore P. W.; Pearcy P.; Meizyte G.; Pickering J.; Mackenzie S. R. An infrared study of CO_2_ activation by holmium ions, Ho^+^ and HoO^+^. Phys. Chem. Chem. Phys. 2022, 24, 22716 10.1039/D2CP02862J. 36106954

[ref36] Iskra A.; Gentleman A. S.; Kartouzian A.; Kent M. J.; Sharp A. P.; Mackenzie S. R. Infrared Spectroscopy of Gas-Phase M^+^(CO_2_)_n_ (M = Co, Rh, Ir) Ion–Molecule Complexes. J. Phys. Chem. A 2017, 121 (1), 133–140. 10.1021/acs.jpca.6b10902. 27992215

[ref37] Becke A. D. Density-functional thermochemistry. III. The role of exact exchange. J. Chem. Phys. 1993, 98 (7), 5648–5652. 10.1063/1.464913.

[ref38] Becke A. D. Density-functional exchange-energy approximation with correct asymptotic behavior. Phys. Rev. A 1988, 38 (6), 3098–3100. 10.1103/PhysRevA.38.3098. 9900728

[ref39] Weigend F. Accurate Coulomb-fitting basis sets for H to Rn. Phys. Chem. Chem. Phys. 2006, 8, 1057–1065. 10.1039/b515623h. 16633586

[ref40] Weigend F.; Ahlrichs R. Balanced basis sets of split valence, triple zeta valence and quadruple zeta valence quality for H to Rn: Design and assessment of accuracy. Phys. Chem. Chem. Phys. 2005, 7 (18), 3297–3305. 10.1039/b508541a. 16240044

[ref41] Grimme S.; Ehrlich S.; Goerigk L. Effect of the damping function in dispersion corrected density functional theory. J. Comput. Chem. 2011, 32 (7), 1456–1465. 10.1002/jcc.21759. 21370243

[ref42] Frisch M. J.; Trucks G. W.; Schlegel H. B.; Scuseria G. E.; Robb M. A.; Cheeseman J. R.; Scalmani G.; Barone V.; Petersson G. A.; Nakatsuji H.; Gaussian 16 Rev. C.01 Gaussian, Inc., Wallingford, Connecticut, U.S., 2016.

[ref43] Addicoat M. A.; Metha G. F. Kick: constraining a stochastic search procedure with molecular fragments. J. Comput. Chem. 2009, 30, 57–64. 10.1002/jcc.21026. 18506695

[ref44] Perdew J. P. Density-functional approximation for the correlation energy of the inhomogeneous electron gas. Phys. Rev. B 1986, 33 (12), 8822–8824. 10.1103/PhysRevB.33.8822. 9938299

[ref45] Fukui K. The path of chemical reactions - the IRC approach. Acc. Chem. Res. 1981, 14 (12), 363–368. 10.1021/ar00072a001.

[ref46] Hratchian H. P.; Schlegel H. B. Ch 10: Finding minima, transition states, and following reaction pathways on ab initio potential energy surfaces. In Theory and Applications of Computational Chemistry: The First Forty Years; Elsevier, 2005.

[ref47] Tolman C. A. The 16 and 18 electron rule in organometallic chemistry and homogeneous catalysis. Chem. Soc. Rev. 1972, 1 (3), 337–353. 10.1039/cs9720100337.

[ref48] Beyer T.; Swinehart D. F. Algorithm 448: number of multiply-restricted partitions. Commun. ACM 1973, 16 (6), 379 10.1145/362248.362275.

[ref49] Barker J. R.; Nguyen T. L.; Stanton J. F.; Aieta C.; Ceotto M.; Gabas F.; Kumar T. J. D.; Li C. G. L.; Preses J. M.; Simmie J. M.; MultiWell-2020 Software; Suite University of Michigan, Ann Arbor, Michigan, U.S., 2020.

[ref50] Shaffer C. J.; Révész Á.; Schröder D.; Severa L.; Teplý F.; Zins E.-L.; Jašíková L.; Roithová J. Can Hindered Intramolecular Vibrational Energy Redistribution Lead to Non-Ergodic Behavior of Medium-Sized Ion Pairs?. Angew. Chem., Int. Ed. 2012, 51 (40), 10050–10053. 10.1002/anie.201203441. 22893525

[ref51] Green A. E.; Schaller S.; Meizyte G.; Rhodes B. J.; Kealy S. P.; Gentleman A. S.; Schollkopf W.; Fielicke A.; Mackenzie S. R. Infrared Study of OCS Binding and Size-Selective Reactivity with Gold Clusters, Au_n_^+^ (n = 1–10). J. Phys. Chem. A 2020, 124 (26), 5389–5401. 10.1021/acs.jpca.0c03813. 32491870

[ref52] Meizyte G.; Green A. E.; Gentleman A. S.; Schaller S.; Schollkopf W.; Fielicke A.; Mackenzie S. R. Free electron laser infrared action spectroscopy of nitrous oxide binding to platinum clusters, Pt_n_(N_2_O)^+^. Phys. Chem. Chem. Phys. 2020, 22 (33), 18606–18613. 10.1039/D0CP02800B. 32785404

[ref53] Hermes A. C.; Hamilton S. M.; Cooper G. A.; Kerpal C.; Harding D. J.; Meijer G.; Fielicke A.; Mackenzie S. R. Infrared driven CO oxidation reactions on isolated platinum cluster oxides, Pt_n_O_m_^+^. Farad. Discuss. 2012, 157, 213–225. 10.1039/c2fd20019h. 23230771

