# Supplementary material for: A Combined Infrared and Computational Study of Gas-Phase Mixed-Ligand Rhodium Complexes: Rh(CO)n(N2O)m+ (n = 1–5, m = 1–4)
Source: J Phys Chem A. 2023 Oct 31;127(44):9220–8. doi: 10.1021/acs.jpca.3c05078 (PMC10641848; doi:10.1021/acs.jpca.3c05078)

A Combined Infrared and Computational Study of Gas-Phase Mixed Ligand Rhodium Complexes: $Rh(CO)_n(N_2O)_m^+$ (n = 1–5, m = 1–4)

*Gabriele Meizyte, Rachael H. Brown, Edward I. Brewer, Peter D. Watson and Stuart R. Mackenzie**

Department of Chemistry, University of Oxford, Physical and Theoretical Chemistry Laboratory, South Parks Road, Oxford, United Kingdom, OX1 3QZ

Supporting Information

Contents:

Table S1: Cartesian coordinates of $Rh(CO)_n(N_2O)_m^+$ complexes. 3
Table S2: Calculated and Experimental Frequencies for $Rh(CO)_n(N_2O)_m^+$ complexes. 14
Table S3: Calculated Complex Binding Energies for $Rh(CO)_n(N_2O)_m^+$ complexes. 16

Figure S1: Calculated vibrational and experimental spectra of the $Rh(CO)_n(N_2O)_m^+$ (n = 1, m = 1-5) complexes. 17
Figure S2: Calculated vibrational and experimental spectra of the $Rh(CO)_n(N_2O)_m^+$ (n = 2-3, m = 2-3) complexes. 18
Figure S3: Internal energy distributions for $Rh(CO)_n(N2O)_m^+$ complexes at 200K and 298K 19

Table S1: Cartesian coordinates of $Rh(CO)_n(N_2O)_m^+$ complexes.

$Rh(CO)(N_2O)^+$

+0.00 eV

N	-1.64701200	0.00057300	0.00037500
N	-2.77008400	-0.00090600	0.00028400
O	-3.92268300	-0.00111000	-0.00028200
C	2.38020700	-0.00131200	-0.00021200
O	3.50228400	-0.00145200	0.00018400
Rh	0.44448000	0.00068200	-0.00005700

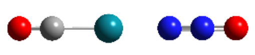

+0.35 eV

N	1.48816300	-0.08883200	-0.00000800
N	2.52113900	0.35485400	0.00000200
O	3.58515600	0.80063300	0.00000400
C	-1.79017700	0.88893000	0.00022000
O	-2.44182800	1.80411300	-0.00010400
Rh	-0.58823700	-0.62297100	-0.00001100

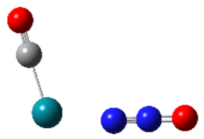

+0.37 eV

N	3.67939200	-0.65632700	-0.00001800
N	2.73025100	-0.08172700	0.00023900
O	1.73173300	0.58980400	0.00063000
C	-2.28979900	-0.18359800	0.00023700
O	-3.40053600	-0.34873300	0.00053000
Rh	-0.39507300	0.09643100	-0.00027200

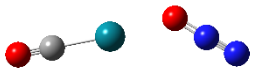

$Rh(CO)_2(N_2O)^+$

+0.00 eV

N	1.73059800	0.30054600	0.00033600
N	2.84708900	0.18067300	-0.00005900
O	3.99424800	0.07120800	-0.00048000
C	-0.57805700	-1.42671300	0.00007800
O	-0.71214200	-2.54646600	-0.00003500
Rh	-0.33492500	0.37600700	0.00014900
C	-2.19959100	0.52066100	-0.00027900
O	-3.32039500	0.61869200	-0.00041700

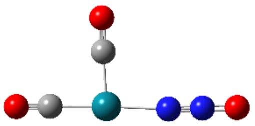

Continued on the next page

+0.34
eV

Rh	0.30181800	-0.39665100	-0.09676900	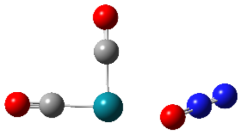
N	-3.64732500	0.19239300	0.62664800	
N	-2.74084900	-0.18681500	0.11278100	
O	-1.79260600	-0.62482000	-0.48982600	
C	2.12426000	-0.41518400	0.21143400	
O	3.23370800	-0.44076000	0.40385500	
C	0.44241600	1.41601000	-0.09509800	
O	0.52581900	2.54123900	-0.10395700	

$Rh(CO)_3(N_2O)^+$

+0.00
eV

Rh	0.32110800	0.00024300	0.00077800	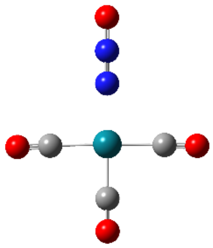
N	-1.76060800	-0.00052500	0.00026900	
N	-2.88184500	-0.00126600	0.00063700	
O	-4.03689000	-0.00211500	0.00113100	
C	0.36145800	1.95845700	-0.00121400	
O	0.39520100	3.07885900	-0.00373200	
C	2.18718000	0.00095400	0.00138200	
O	3.31292900	0.00127600	0.00197600	
C	0.36425900	-1.95830600	-0.00131900	
O	0.40000300	-3.07864600	-0.00368100	

+0.38
eV

Rh	-0.27808400	0.00133000	-0.10696700	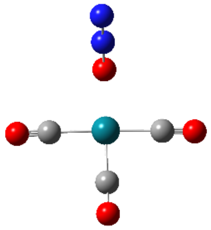
N	3.65598000	-0.05134900	0.81776600	
N	2.77168500	-0.07968100	0.14755700	
O	1.85142400	-0.11623000	-0.62743700	
C	-0.40911400	-1.95520700	-0.09121300	
O	-0.49719400	-3.07257300	-0.08264600	
C	-0.22176400	1.95970000	-0.10957700	
O	-0.20365000	3.08061000	-0.11224300	
C	-2.07875700	0.09007900	0.27928500	
O	-3.17833900	0.14443500	0.52048600	

Continued on the next page

$Rh(CO)_4(N_2O)^+$

+0.00 eV

N	-2.68282000	-0.00012900	0.38037800
N	-3.69713500	-0.00002400	0.86390400
O	-4.74938100	0.00009400	1.36627900
C	1.87744500	1.38055500	0.69512200
O	2.49559900	2.17361300	1.19057700
Rh	0.80690200	-0.00000400	-0.17667100
C	-0.23882600	1.38382400	-1.09280500
O	-0.82784200	2.17563200	-1.62278500
C	1.87727400	-1.38066500	0.69515300
O	2.49531400	-2.17376400	1.19068600
C	-0.23881600	-1.38374000	-1.09295100
O	-0.82786000	-2.17540000	-1.62312100

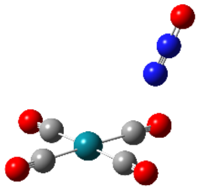

$Rh(CO)_5(N_2O)^+$

+0.00 eV

C	-2.46067400	1.13913700	-0.00039800
O	-3.39415700	1.76558400	-0.00042200
Rh	-0.81483100	0.03086200	-0.00028200
C	0.24645700	1.65959600	-0.00155800
O	0.84289300	2.60865700	-0.00258800
C	0.09613100	-0.56835600	-1.68919900
O	0.61400900	-0.89969300	-2.62875400
C	-1.91940300	-1.56759600	0.00059700
O	-2.55817600	-2.48947500	0.00086200
C	0.09523000	-0.56544300	1.69055300
O	0.61243500	-0.89523700	2.63100800
N	2.97362100	0.03145400	0.00070000
N	4.08786400	-0.11326700	0.00051700
O	5.24431300	-0.26485200	0.00042100

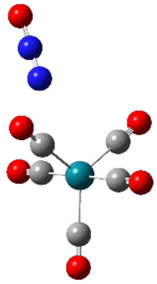

Continued on the next page

+0.33 eV

C	-0.07098700	1.36054700	1.37577000	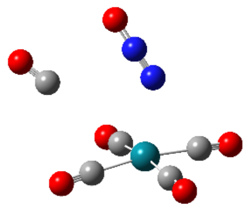
O	-0.56726400	1.97951000	2.16877000	
Rh	0.84357300	0.32481700	0.00107300	
C	-3.16788900	0.75310500	0.00321900	
O	-4.28273500	0.86565900	-0.00234000	
C	1.96268900	-0.50334900	1.38000100	
O	2.61933800	-0.95713400	2.16719700	
C	1.96272200	-0.49363300	-1.38364400	
O	2.61932300	-0.94196800	-2.17399000	
C	-0.07057700	1.37056500	-1.36627500	
O	-0.56661200	1.99529800	-2.15489900	
N	-0.68683700	-2.07606500	-0.00955800	
N	-1.76470500	-2.39444100	-0.00656200	
O	-2.88401700	-2.72219200	-0.00347300	

$Rh(CO)(N_2O)_2^+$

+0.00 eV

Rh	0.00010900	-0.32396400	0.00073800	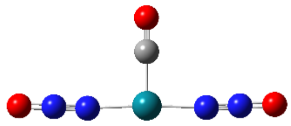
N	-1.97964000	-0.39417500	0.00010900	
N	-3.10057300	-0.33914400	-0.00073300	
O	-4.25667200	-0.30480800	-0.00167800	
N	1.97974000	-0.39430400	0.00048800	
N	3.10065500	-0.33892000	-0.00071600	
O	4.25673300	-0.30435700	-0.00184300	
C	-0.00017000	1.47659000	0.00029800	
O	-0.00070500	2.60724400	-0.00010800	

+0.29 eV

Rh	0.07456800	0.71154700	0.00000300	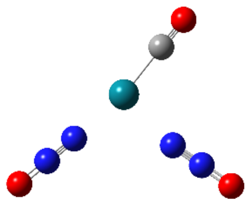
N	0.83393200	-1.41753800	-0.00107000	
N	1.41463500	-2.37907900	-0.00021700	
O	2.00453300	-3.37576100	0.00064200	
N	-1.96220500	0.09433400	0.00004700	
N	-3.04621800	-0.19737900	0.00007500	
O	-4.16168700	-0.49877800	0.00012700	
C	1.79605800	1.59361000	0.00010500	
O	2.80554200	2.08908300	0.00015500	

Continued on the next page

+0.48 eV

Rh	-0.00009900	-0.53134500	-0.63452600
N	-1.66612000	0.75196600	-0.09709000
N	-2.57371700	1.29178600	0.28606500
O	-3.50693400	1.85443500	0.67500100
N	1.66476500	0.75386000	-0.09741700
N	2.57210700	1.29412600	0.28569900
O	3.50538100	1.85708000	0.67408100
C	0.00197400	-2.00027500	0.62893100
O	0.00322500	-2.80276300	1.41832800

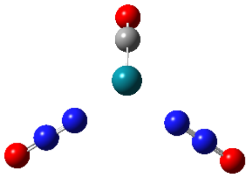

+0.49 eV

Rh	0.03837800	-0.36611100	0.16030500
N	3.83470000	0.00100900	-0.86083600
N	2.97184800	-0.30284300	-0.23377600
O	2.08149200	-0.65854400	0.50172200
N	-1.90661900	-0.34694600	-0.08920100
N	-3.01312400	-0.23344100	-0.23699900
O	-4.15731900	-0.14166200	-0.38885600
C	0.09940900	1.42850500	0.13733200
O	0.13444400	2.56014400	0.12563200

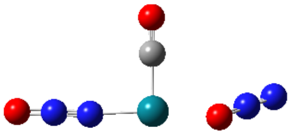

$Rh(CO)(N_2O)_3^+$

+0.00 eV

Rh	-0.00003500	0.27265600	0.00015400
C	-0.00018000	2.11842900	-0.00019500
O	-0.00026000	3.24759700	-0.00035100
N	-1.98892100	0.27009500	0.00016200
N	-3.10691700	0.34676400	-0.00003100
O	-4.26521300	0.41218800	-0.00022000
N	0.00004400	-1.82391300	0.00045800
N	0.00018700	-2.94482600	-0.00006400
O	0.00044500	-4.10239600	-0.00046500
N	1.98891800	0.27035000	0.00008000
N	3.10689900	0.34717700	-0.00003500
O	4.26517900	0.41266000	-0.00018400

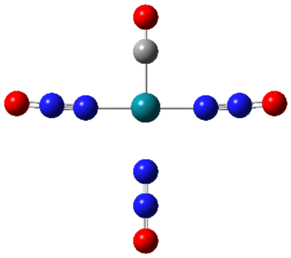

Continued on the next page

+0.30 eV

Rh	-0.16312500	0.19229100	0.00107900
C	-0.49717600	1.98143100	-0.00785300
O	-0.70225400	3.09373700	-0.01309300
N	1.79070100	0.53654700	0.00352100
N	2.85771300	0.88497100	0.00547400
O	3.96707700	1.22164800	0.00744900
N	-2.11031900	-0.20458600	0.00011900
N	-3.21751200	-0.37776400	0.00113000
O	-4.36100300	-0.56911400	0.00303500
N	2.10718800	-3.17689000	-0.02223800
N	1.16228900	-2.59304700	-0.00417500
O	0.12034000	-1.99955600	0.01657900

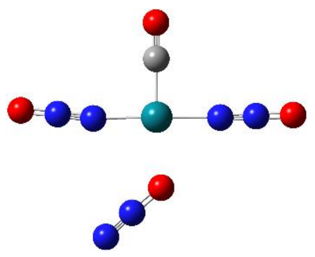

+0.30 eV

Rh	0.00197600	0.23856000	0.07728900
C	0.00594800	2.01317200	-0.33182100
O	0.00831200	3.11376000	-0.59175400
N	-1.98299500	0.21606600	0.09471500
N	-3.10035200	0.30407000	0.12577700
O	-4.25728800	0.37681000	0.15663400
N	-0.02514700	-3.67884200	-0.86422200
N	-0.01398300	-2.81021900	-0.17209400
O	-0.00162400	-1.90576800	0.61831100
N	1.98684200	0.20669500	0.09342800
N	3.10471000	0.28780100	0.12406400
O	4.26208500	0.35354800	0.15446700

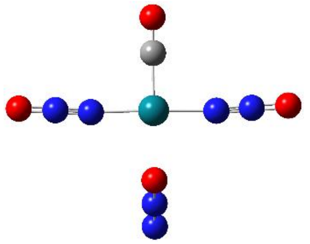

+0.49 eV

Rh	0.09272900	-0.18057500	-0.10745000
C	0.60300500	-1.94532700	-0.20328400
O	0.91532100	-3.03051300	-0.26721200
N	-1.77847900	-0.74392300	0.09776900
N	-2.82119500	-1.13704400	0.21422300
O	-3.90759500	-1.52979100	0.33558400
N	-0.47230900	1.83443200	-0.02945200
N	-0.76139400	2.91743800	-0.01807500
O	-1.05965900	4.03567900	-0.00409800
N	3.95151200	-0.20705400	0.73891800
N	3.03170200	0.13644200	0.22090900
O	2.07197300	0.54909900	-0.37866000

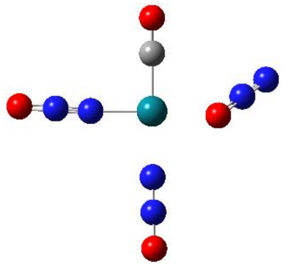

Continued on the next page

$Rh(CO)(N_2O)_4^+$

+0.00 eV

Rh	-0.98891700	-0.31793200	-0.00012400
O	-4.54126200	2.04741900	0.00096800
N	-3.56280500	1.42324900	0.00069500
N	-2.61178000	0.83082200	0.00044700
O	1.65676300	3.15696600	-0.00136600
N	0.93063300	2.25514100	-0.00096400
N	0.22335900	1.38695000	-0.00059000
O	5.69648300	0.64534100	0.00186400
N	4.53742700	0.51516700	0.00081500
N	3.42084100	0.38972000	-0.00012900
O	2.49807000	-2.77469600	-0.00052700
N	1.55035700	-2.10460200	-0.00052300
N	0.64074000	-1.45247600	-0.00052300
C	-2.04983700	-1.82860900	0.00015600
O	-2.69769300	-2.75368000	0.00031400

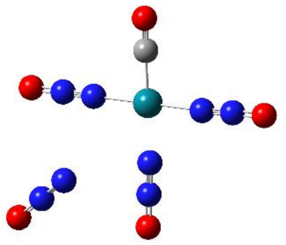

+0.30 eV

Rh	-0.86906500	-0.28734000	-0.10368900
O	0.05941500	1.65215000	-0.60287700
N	0.99092500	1.82591900	-1.33755200
N	1.84984700	2.02901400	-2.01140200
O	2.73123000	-2.47287700	-0.71906000
N	1.75061000	-1.86651100	-0.59145700
N	0.80935000	-1.26857900	-0.48014400
O	2.90873200	0.57856000	0.73139700
N	3.90812200	0.86497500	1.30406900
N	4.84629900	1.13459200	1.84436000
O	-4.44559800	1.87565800	0.75002700
N	-3.46822700	1.29880200	0.51005100
N	-2.51945500	0.75125700	0.27411900
C	-1.70913600	-1.84156700	0.33275300
O	-2.22997800	-2.80931500	0.60115700

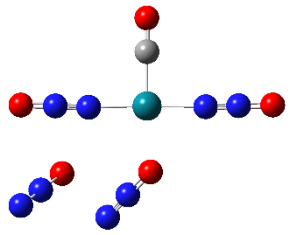

Continued on the next page

+0.49 eV

Rh	0.52779600	-0.39608800	0.41291400
O	-3.01559000	1.23765100	-3.15739000
N	-2.62086900	0.88976900	-2.11583100
N	-2.23737300	0.55690600	-1.11344500
O	4.03979800	0.76726000	-1.66834900
N	3.07327600	0.47084300	-1.09450700
N	2.13901400	0.19926100	-0.53890400
O	-0.36815900	3.62871700	1.86789500
N	-0.10266800	2.57374000	1.46980400
N	0.15580400	1.55474200	1.08227000
O	-1.16991200	-0.95976900	1.56859000
N	-2.12774400	-1.52486500	1.10913400
N	-3.03330300	-2.05107800	0.74208300
C	0.85812500	-2.12351600	-0.11933800
O	1.06104300	-3.18887900	-0.44191000

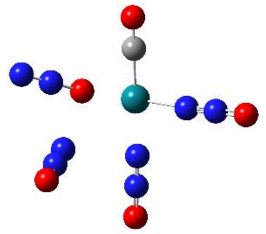

$Rh(CO)_2(N_2O)_2^+$

+0.00 eV

Rh	-0.00009200	0.41796300	0.00150300
C	-1.29049300	1.75843800	-0.00195200
O	-2.07745300	2.56493700	-0.00785500
C	1.28474700	1.76384500	0.00202300
O	2.06765200	2.57426900	0.00244600
N	1.47120700	-1.06267200	0.00083000
N	2.32002800	-1.79521400	-0.00215700
O	3.18868300	-2.55784100	-0.00488400
N	-1.46576200	-1.06825500	0.00146400
N	-2.31480800	-1.80054800	0.00111100
O	-3.18338700	-2.56326600	0.00069200

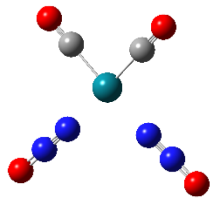

+0.32 eV

Rh	-0.05477800	-0.31458900	-0.00010400
C	-1.68738400	-1.20037700	-0.00017600
O	-2.68249700	-1.73149700	-0.00024100
C	0.73820300	-1.97217400	0.00026800
O	1.23553300	-2.98484100	0.00049000
N	1.78208000	0.66744500	-0.00010500
N	2.79357400	1.15114100	-0.00001200
O	3.83256400	1.65627700	0.00012900
N	-3.08867400	2.40862200	0.00084000
N	-2.03755200	2.05191500	0.00012000
O	-0.88383500	1.71480300	-0.00059900

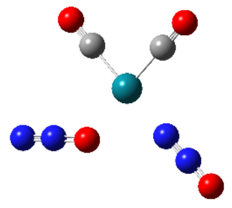

Continued on the next page

+0.48 eV

Rh	-0.00002600	0.00007700	-0.00016700
C	-0.00051800	-1.95356300	-0.00046500
O	-0.00045000	-3.07584100	-0.00079400
C	0.00041800	1.95378300	-0.00049400
O	0.00083700	3.07605800	-0.00077900
N	1.99231500	-0.00028900	0.00041100
N	3.11276400	-0.00054400	0.00064600
O	4.27195800	-0.00056800	0.00093300
N	-1.99237500	0.00057700	-0.00056500
N	-3.11282400	-0.00001900	0.00053700
O	-4.27202000	-0.00000800	0.00140100

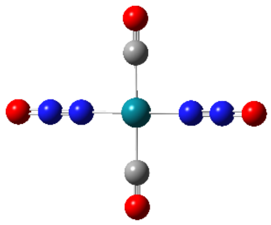

$Rh(CO)_2(N_2O)_3^+$

+0.00 eV

Rh	1.27728200	0.00080800	-0.00079900
C	2.62014700	1.28892800	0.00495600
O	3.42822900	2.07446700	0.00866700
C	2.61825900	-1.28921400	0.00276900
O	3.42504200	-2.07609500	0.00488700
N	-0.21107400	1.45649000	-0.00480500
N	-1.02049800	2.23032100	-0.00690700
O	-1.85793700	3.02791700	-0.00909700
N	-0.21227200	-1.45357300	-0.00568500
N	-1.01831000	-2.23092900	-0.00579800
O	-1.85280800	-3.03161700	-0.00615600
N	-3.21039600	-0.00094500	0.00442700
N	-4.33425200	-0.00039600	0.00734200
O	-5.50009000	0.00015300	0.01039900

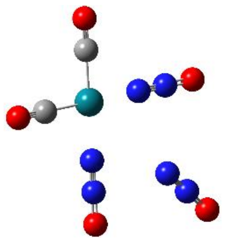

Continued on the next page

+0.30 eV

Rh	0.46306800	0.64254400	0.13560200	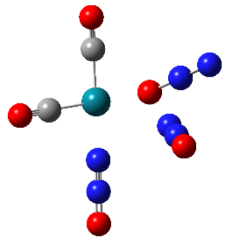
C	1.53885700	1.18699500	-1.24715500	
O	2.20646500	1.51183500	-2.09787100	
C	-0.51452500	2.19838900	-0.12953800	
O	-1.11416800	3.14217300	-0.27914100	
N	-1.69759300	-1.35862700	-0.72378200	
N	-2.10709900	-1.80194500	-1.67207400	
O	-2.52818600	-2.26140100	-2.65706200	
N	-2.96521900	-0.22160200	2.11523600	
N	-1.86879000	-0.09443000	1.99883100	
O	-0.67744900	0.03180000	1.92202300	
N	1.60982600	-1.06412900	0.48376400	
N	2.25575300	-1.96065200	0.67143200	
O	2.91681300	-2.88904100	0.86758000	

+0.49 eV

Rh	0.79520600	0.24621100	0.13873900	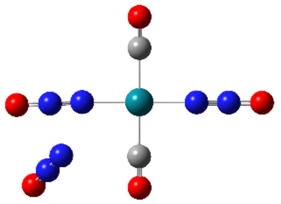
C	1.72039700	1.80873700	-0.57674900	
O	2.25224500	2.70871700	-0.98586600	
C	-0.12689300	-1.31402200	0.86981300	
O	-0.63930500	-2.21559600	1.29866400	
N	-3.18302000	-0.69885700	-0.37005400	
N	-4.13094600	-1.19691800	-0.70917700	
O	-5.11461800	-1.71606400	-1.06240600	
N	-0.84982800	1.33514300	0.39455000	
N	-1.82006800	1.87927800	0.51764800	
O	-2.82096200	2.44972500	0.64716500	
N	2.43838700	-0.84940400	-0.11884600	
N	3.36077700	-1.46837800	-0.26478900	
O	4.31609000	-2.10851300	-0.41592600	

Continued on the next page

$Rh(CO)_3(N_2O)_2^+$

+0.00 eV

Rh	1.04716400	-0.05909400	0.10526100	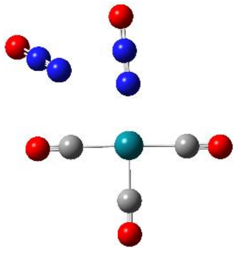
N	-0.39911800	1.38171900	0.48477800	
N	-1.27641400	2.05929400	0.64523300	
O	-2.17508800	2.76556100	0.81342200	
C	2.24305900	1.27950600	-0.67494900	
O	2.93616200	2.03909500	-1.12178200	
C	2.33921500	-1.35975000	-0.23621800	
O	3.11785100	-2.14669100	-0.44327600	
C	-0.08767300	-1.45266800	0.88473700	
O	-0.71578600	-2.26360700	1.33668000	
N	-2.93278300	-0.31210800	-0.38078100	
N	-3.92627900	-0.67722600	-0.75776700	
O	-4.95662100	-1.05749200	-1.14984800	

+0.33 eV

Rh	0.70561600	0.26551900	-0.07289600	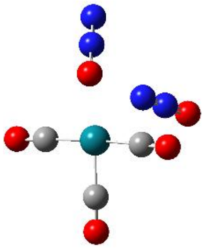
N	-2.98523300	1.98119000	-0.46168700	
N	-1.90815000	1.82633200	-0.68049200	
O	-0.74557400	1.69769200	-0.95045900	
C	1.04345700	-0.43959100	-1.87056000	
O	1.25007100	-0.83232500	-2.90024500	
C	0.53760800	1.04030900	1.71302100	
O	0.45834600	1.49581100	2.73537000	
C	2.10339700	-0.75432600	0.54926900	
O	2.95971100	-1.38136000	0.93235200	
N	-1.87763700	-1.00654300	0.16599700	
N	-2.29433600	-2.01015100	0.45528800	
O	-2.72280200	-3.05013200	0.75501000	

Table S2: Calculated and Experimental Frequencies for $Rh(CO)_n(N_2O)_m^+$ complexes**.**

n+m = 2	Rel. Eng.	Calculated	Experimental
$^3Rh(CO)(N_2O)^+$	0.00	2170 (556), 2308 (742)	2153, 2181, 2213, 2258
	0.35	2151 (451), 2285 (705)	
	0.37	2165 (460), 2253 (328)	

n+m = 3

$^1Rh(CO)(N_2O)_2^+$	0.00	2112 (438),<br>2313 (1478), 2322 (<5)	2101, 2300
	0.49	2100 (429),<br>2240 (389), 2321 (602)	
$^3Rh(CO)(N_2O)_2^+$	0.29	2146 (613), 2272 (759), 2299 (635)	
	0.48	2130 (517), 2276 (967), 2285 (416)	
$^1Rh(CO)_2(N_2O)^+$	0.00	2118 (534), 2167 (489), 2310 (817)	2104, 2158, 2269
	0.34	2110 (540), 2164 (363), 2252 (318)	

n+m = 4

$^1Rh(CO)(N_2O)_3^+$	0.00	2120 (664),<br>2307 (674), 2321 (1231), 2329 (37)	2103, 2227, 2302
	0.30	2110 (563),<br>2245 (362), 2310 (912), 2325 (373)	
	0.30	2112 (555),<br>2243 (363), 2316 (1273), 2323 (8)	
	0.49	2106 (645),<br>2239 (434), 2308 (747), 2328 (475)	
$^1Rh(CO)_2(N_2O)_2^+$	0.00	2118 (643), 2160 (623),<br>2307 (911), 2319 (562)	2113, 2147, 2161, 2291
	0.32	2108 (611), 2154 (514),<br>2248 (412), 2315 (745)	
	0.48	2131 (991), 2191 (0),<br>2327 (1259) 2332 (0)	
$^1Rh(CO)_3(N_2O)^+$	0.00	2140 (554), 2148 (915), 2208 (75),<br>2318 (728)	2142, 2162, 2286
	0.38	2133 (487), 2146 (910), 2208 (46),<br>2246 (327)	

Continued on the next page

n+*m* = 5

$^{1}Rh(CO)(N_2O)_4^+$	0.00	2119 (705), 2231 (642),<br>2332 (613), 2324 (1033), 2335 (183)	2106, 2118,<br>2117, 2233, 2333
	0.30	2109 (594), 2237 (479),<br>2248 (310), 2320 (1206) 2326 (19)	
	0.49	2103 (670), 2228 (614),<br>2242 (364), 2308 (736), 2327 (452)	
$^{1}Rh(CO)_2(N_2O)_3^+$	0.00	2117 (650), 2159 (702),<br>2230 (590), 2314 (728), 2328 (609)	2121, 2138, 2163,<br>2233, 2342
	0.30	2104 (620), 2151 (539),<br>2229 (633), 2250 (356), 2314 (715)	
	0.49	2130 (1033) 2191 (0),<br>2232 (604), 2328 (970), 2337 (242)	
$^{1}Rh(CO)_3(N_2O)_2^+$	0.00	2137 (581), 2147 (955), 2208 (75),<br>2232 (581), 2324 (688)	2141, 2158, 2212,<br>2232, 2237
	0.33	2124 (502), 2141 (904), 2203 (68),<br>2231 (654), 2250 (301)	
$^{1}Rh(CO)_4(N_2O)^+$	0.00	2153 (880), 2153 (943),<br>2183 (7), 2227 (3),<br>2231 (608)	2161, 2232

n+*m* = 6

$^{1}Rh(CO)_5(N_2O)^+$	0.00	2133 (720), 2141 (572), 2155 (532),<br>2158 (266), 2214 (<5), 2231 (602)	2149, 2177, 2212, 2232
	0.33	2147 (873), 2147 (915), 2174 (8),<br>2178 (46), 2220 (46), 2225 (583)	

Table S3: Calculated Complex Binding Energies for $Rh(CO)_n(N_2O)_m^+$ complexes. Binding energies are reported in eV for loss channels of –N_2O (purple), –CO (orange), –2xN_2O (green), –[CO, N_2O] (pink) and –2xCO (mustard).

		n				
		1	2	3	4	5
m	1	1.25<br>1.96	1.31<br>1.72	1.23<br>1.69	0.17<br>0.79<br>2.02	0.17<br>0.46<br>0.63<br>1.25
	2	0.82<br>1.50	1.12<br>2.01	0.17<br>1.19<br>1.29	0.17<br>0.75<br>1.86<br>1.40	
	3	0.99<br>1.99<br>1.81				
	4	0.17<br>1.20<br>1.16				

Figure S1: Calculated low-lying isomers within +0.5 eV from the putative ground state and their vibrational analysis as well as experimental spectra of the $Rh(CO)_n(N_2O)_m^+$ (n = 1, m = 1-5) species presented as the absolute difference in enhancement signal for all observed daughter channels. The band positions in red are due to the vibrations of CO ligand whereas N_2O vibrations are in blue. The dashed red and blue lines denote the experimental values of free CO (2143.2 cm^{-1}) and N=N in the free N_2O (2223.5 cm^{-1}) stretches, respectively. In some cases, the spectra are enlarged by ten times, as indicated**.**

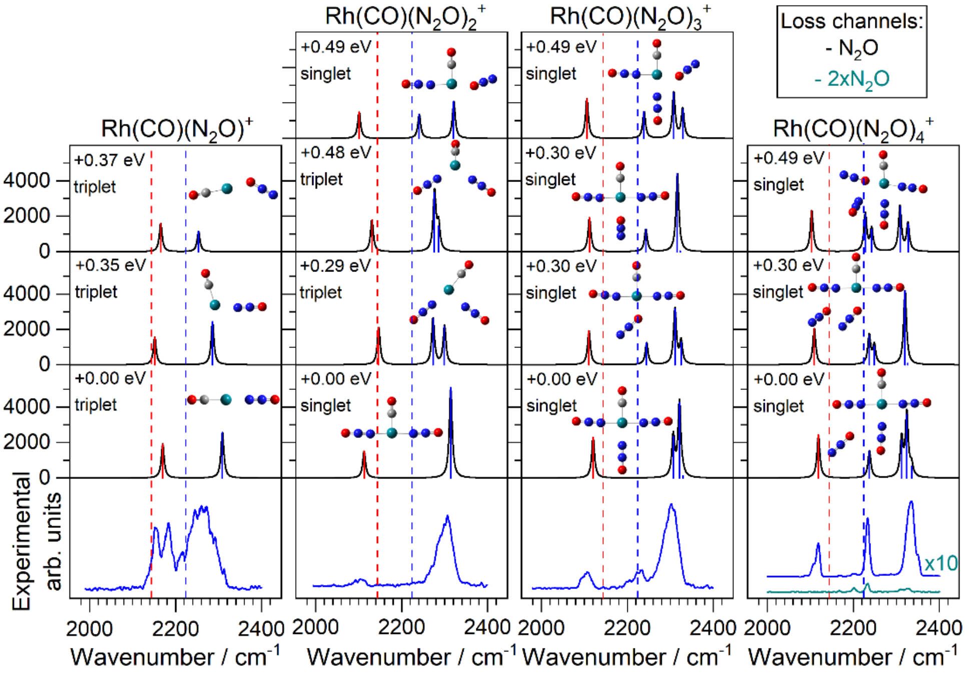

Figure S2: Calculated low-lying isomers within +0.5 eV from the putative ground state and their vibrational analysis as well as experimental spectra of the $Rh(CO)_n(N_2O)_m^+$ (n = 2-3, m = 2-3) species presented as the absolute difference in enhancement signal for all observed daughter channels. The band positions in red are due to the vibrations of CO ligands whereas N_2O vibrations are in blue. The dashed red and blue lines denote the experimental values of free CO (2143.2 cm^{-1}) and N=N in the free N_2O (2223.5 cm^{-1}) stretches, respectively.

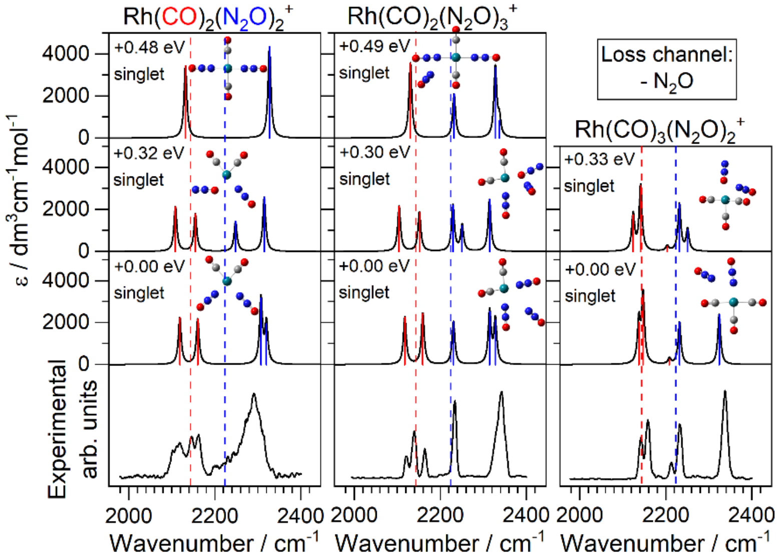

Figure S3: Internal energy distributions for the putative ground state geometries of the $Rh(CO)_{2\text{-}3}(N_2O)_{2\text{-}3}^+$ (top panel), $Rh(CO)(N_2O)_{1\text{-}4}^+$ (middle panel), and $Rh(CO)_{1\text{-}5}(N_2O)^+$ (bottom panel) species at 200K (in purple) and 298K (in orange), with dashed lines showing the distributions upon absorbing a photon of an average energy of $h\nu$ = 0.28 eV. The calculated binding energies for different adsorbates are indicated.

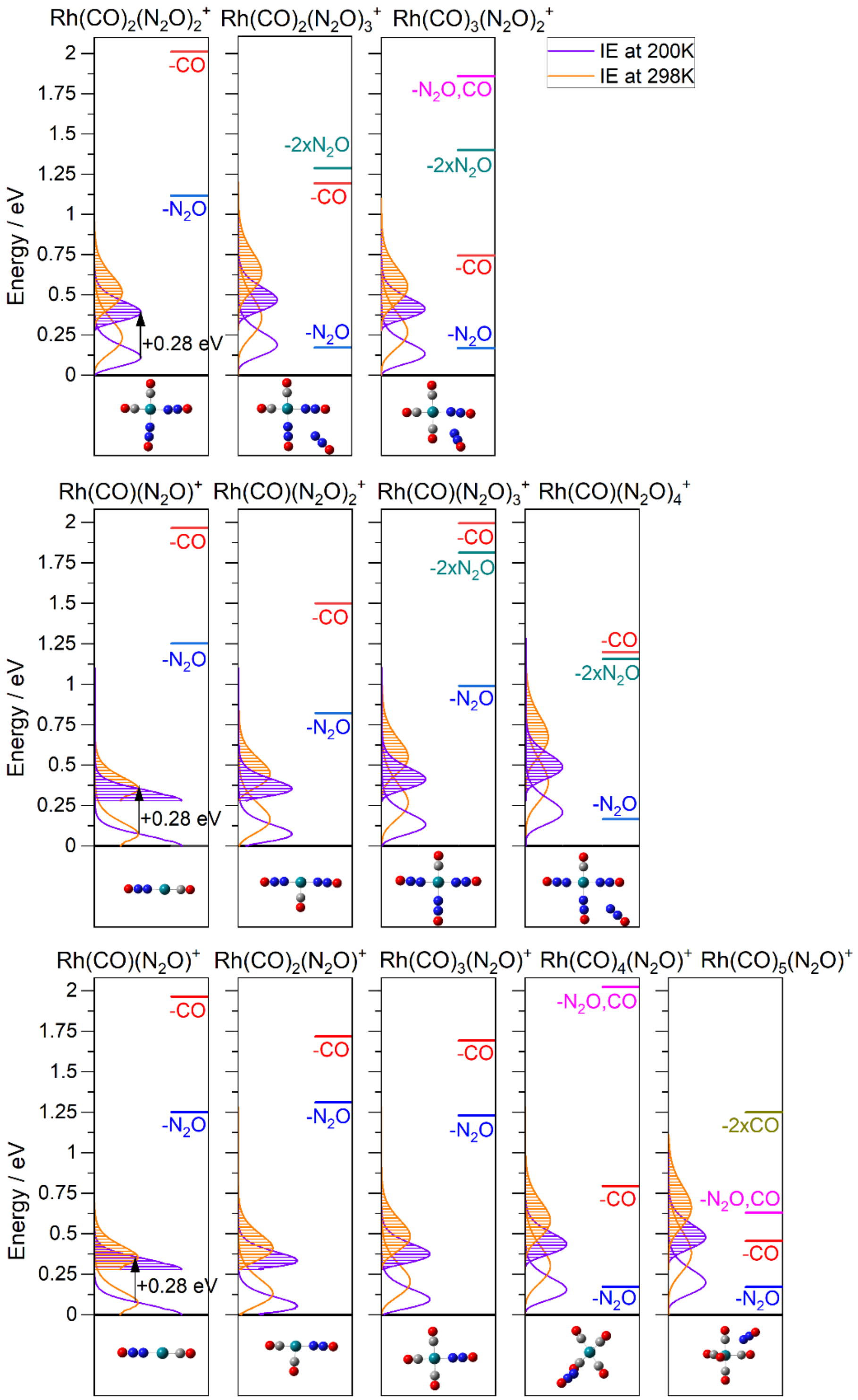

Supplement: Supplementary file 1 — jp3c05078_si_001.pdf [file jp3c05078_si_001.pdf]
